# Evaluating the feasibility and preliminary efficacy of a Cognitive Occupation-Based programme for people with Multiple Sclerosis (COB-MS): protocol for a feasibility cluster-randomised controlled trial

**DOI:** 10.1186/s13063-020-4179-5

**Published:** 2020-03-17

**Authors:** Christopher P. Dwyer, Alberto Alvarez-Iglesias, Robert Joyce, Timothy J. Counihan, Dympna Casey, Sinéad M. Hynes

**Affiliations:** 1 Discipline of Occupational Therapy, School of Health Sciences, National University of Ireland Galway, Galway, Ireland; 2 Health Research Board Clinical Research Facility, National University of Ireland Galway, Galway, Ireland; 3 Department of Neurology, University Hospital Galway, Galway, Ireland; 4 Qualitative Research Trials Centre (QUESTS), School of Nursing & Midwifery, National University of Ireland Galway, Galway, Ireland

**Keywords:** Multiple sclerosis, Occupational therapy, Cognitive occupation-based programme, Cognitive rehabilitation, Feasibility, Protocol

## Abstract

**Background:**

Cognitive difficulties experienced by people with multiple sclerosis (MS) impact their quality of life and daily functioning, from childcare and work, to social and self-care activities. Despite the high prevalence of cognitive difficulties seen in MS, there is a lack of developed programmes that target cognition, while also supporting patients by helping them to function well in everyday life. The Cognitive Occupation-Based programme for people with MS (COB-MS) was developed as a holistic, individualised cognitive rehabilitation intervention. It addresses the wide-ranging symptoms and functional difficulties that present in MS, including the ability to maintain employment, social activities, home management and self-care. The aim of the current research is to evaluate the feasibility and preliminary efficacy of COB-MS for people with MS. The focus is on feasibility outcomes as well as functioning associated with cognitive difficulty and secondary outcomes related to cognition, fatigue and quality of life.

**Methods:**

One hundred and twenty people with MS will be assigned to participate in either the COB-MS programme or a treatment as usual, wait-list control group as part of this single-blind, cluster-randomised controlled feasibility and preliminary efficacy trial of the COB-MS programme. The COB-MS group will participate in an eight-session occupational-based cognitive rehabilitation programme over 9 weeks. The primary outcome measure is the goal attainment scaling at 12 weeks. Participants will be assessed pre-intervention, post-intervention and at 12 weeks post-intervention and 6 months post-intervention. Qualitative evaluations of participants’ perspectives will also be examined as part of the feasibility study.

**Discussion:**

Results will provide recommendations for a future definitive trial of COB-MS, with respect to both feasibility and preliminary, clinical efficacy. In the event that results indicate efficacy, study findings will suggest that COB-MS requires consideration as a means of enhancing cognitive and daily functioning in people living with MS.

**Trial registration:**

ISRCTN: ISRCTN11462710. Registered on 9 September 2019.

## Background

Approximately 40–65% of people living with multiple sclerosis (MS) have difficulties with cognition [1–3]. With an average onset age of 30 years [4] and an unpredictable course (e.g. with respect to development and typology; see [5]), MS and its associated cognitive difficulties can have wide-ranging impacts on quality of life and daily functioning in equally wide-ranging occupations, from childcare and work, to social and self-care activities. Research has found that 90% of the estimated 9000 people in Ireland living with MS are of working age [6] and that both fatigue and cognitive difficulties in MS are associated with an increased likelihood of being unemployed [7]. Cognitive difficulties associated with MS typically affect the domains of information processing [8, 9]; memory [10], including working memory impairments [11]; attention (divided and sustained) [12]; new learning [13]; problem-solving [14]; and language (verbal fluency and naming) [15, 16]. The existence of such heterogeneous cognitive difficulties suggests that an individualised approach to rehabilitation may suit this population best. Furthermore, in order to address the needs of people with MS who are not working, there should be access to interventions that support people in working and living independently [6]. Despite the high prevalence of cognitive problems experienced by people with MS, very little has been done to address these difficulties and, more importantly, the impact that they have on a person’s everyday life.

Cognitive abilities have been shown to correlate with other symptoms and functional difficulties that present in MS, including fatigue and depression, as well as the ability to maintain employment, social activities, managing the home and self-care [17–20]. These difficulties impact all aspects of life and clearly suggest an urgent need for a targeted approach to cognitive rehabilitation intervention. A recent, small-scale study [21], with similar aims and objectives, examined the efficacy of a cognitive occupation-based programme for people with MS (COB-MS) via pre-/post-test design. Results from 12 participants revealed significant improvements in relation to goal attainment, memory and attention—indicating a need for further research. Subsequent qualitative research on COB-MS revealed that the COB-MS intervention was perceived as useful and feasible; specifically, people with MS reported that the programme was a validating intervention, and occupational therapists noted its client-centred nature [22]. Similarly, a recent meta-synthesis of qualitative studies, examining the perspectives of people with MS towards cognitive rehabilitation, found that participation in such rehabilitation facilitated reflection and awareness of cognitive deficits; enhanced understanding of MS; provided emotional and social improvements; increased cognitive strategy use; and enhanced confidence, perseverance and optimism [23]. However, to date, there remains a paucity of evidence, as few methodologically rigorous research programmes have been developed to reduce impairment in cognition, decrease the detrimental effects of impairment and support patients by helping them to function well in everyday life [24–26].

COB-MS was developed to provide holistic cognitive rehabilitation in MS [22]. COB-MS focuses on rehabilitation through an individualised cognitive intervention, measured by and taught through an occupational participation perspective. That is, participants have access to a non-pharmacological treatment for cognition that focuses on the aspects of their own daily life that are important to them.

## Aims and objectives

The aim of the current research is to evaluate the feasibility and preliminary efficacy of COB-MS on cognitive and daily functioning for people with MS. Specifically, this study’s objectives are to:

1. Assess the integrity of the protocol and field test the outcome measures and procedures used in the trial
2. Determine the preliminary efficacy of COB-MS in comparison with treatment as usual
3. Determine the acceptability of COB-MS and investigate the barriers and facilitators to using COB-MS
4. Determine the appropriateness of progression to a definitive trial.

## Methods

### Design

The current study is a single-blind, cluster-randomised controlled feasibility trial of COB-MS (see “Interventions”). The structure is based on the Medical Research Council’s (MRC’s) “Framework for development and evaluation of RCTs for complex interventions to improve health” [27]. This study will use a treatment as usual, wait-list control group design and a pre-post study design with two additional follow-up testing times: 12 weeks and 6 months follow-up (i.e. four data points). Follow-up data will be collected to evaluate the sustainability of intervention gains, if evident, as well as to gather data on retention over the entire duration of the trial. Fidelity and qualitative data will also be collected to inform the feasibility assessment of this trial.

### Participants

#### Setting

This trial is a community-based research study that will run COB-MS groups at various locations across the Republic of Ireland. Participants’ own homes and accessible community venues will be used as locations to run the intervention and collect data. The main study site is at the National University of Ireland (NUI) Galway; but data will be collected nationwide, dependent on the location of the participants.

#### Sample size

**Fig. 1:**
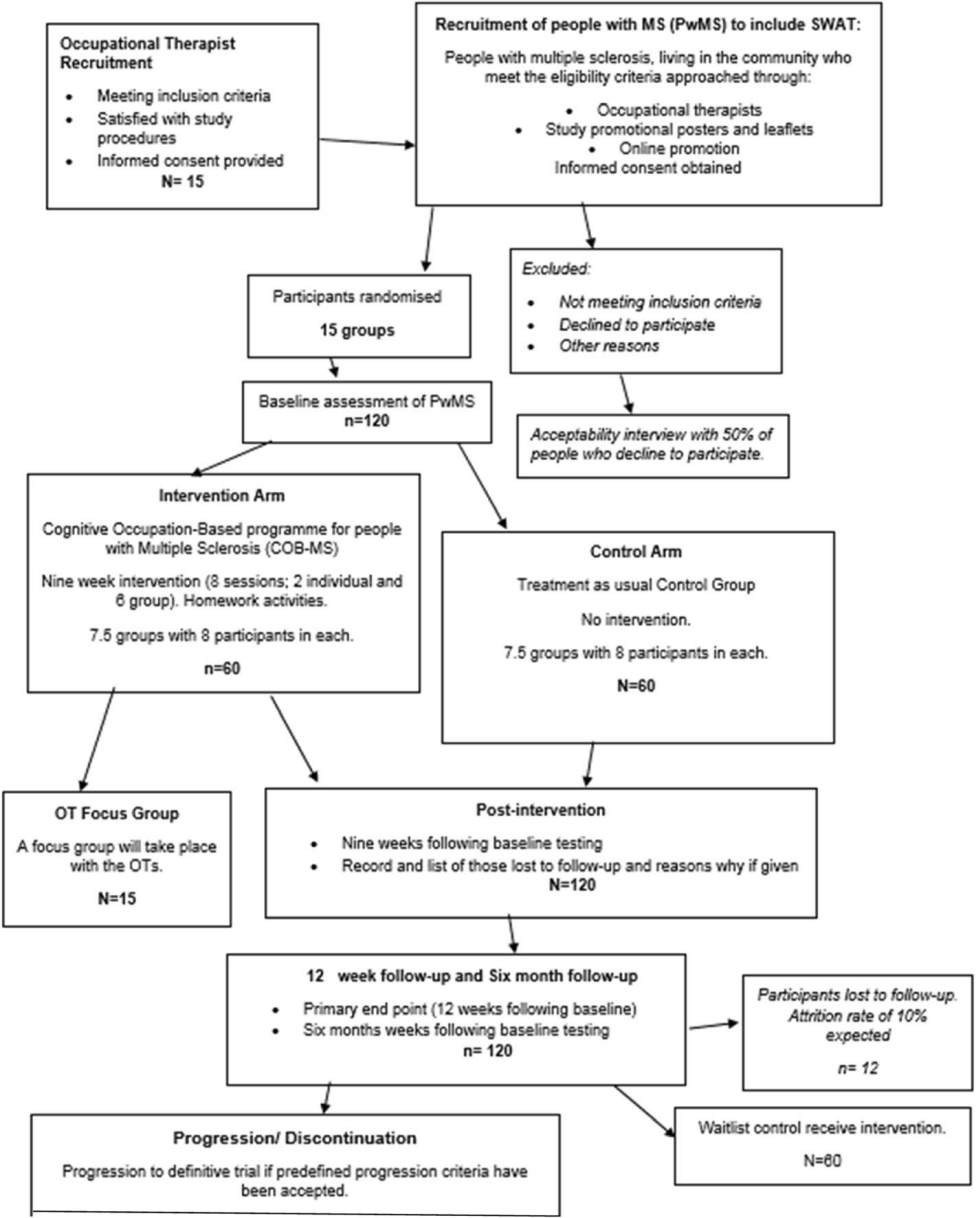
COB-MS CONSORT flowchart of study participants

As this is a feasibility study, a formal sample size calculation to evaluate the clinical effectiveness of COB-MS is not required. Instead, a pragmatic approach is adopted. It is aimed at examining the rate of retention of participants during the intervention and follow-up periods and is based on an average recruitment rate for National Institute for Health Research [28]-funded randomised controlled trials (RCTs). A 9% attrition rate is likely to occur [26]; therefore, if this were an individually randomised trial (at the patient level), a sample of 90 participants would allow for estimation of a retention rate of 91% with a 95% confidence interval (CI) of width equal to 13%. After allowing for clustering, assuming 8 participants per cluster (occupational therapist) and an intracluster correlation coefficient (ICC) = 0.05, the sample size becomes 90 × [1 + (8–1) × 0.05] = 121. Thus, the number of occupational therapists needed is 121/8 = 15. A final sample size of 15 × 8 = 120 people with MS is deemed large enough to provide information regarding the practicalities of a potential definitive randomised trial. This follows Consolidated Standards of Reporting Trials (CONSORT) guidelines for sample size calculation in feasibility studies. See Fig. 1 for the CONSORT flowchart of study participants.

#### Gender issues

A number of different gender issues are relevant to this trial because of the condition (MS) under study, including the ratio of prevalence, age of onset, disease progression, ability to cope and quality of life issues. Data on gender will be collected and will be accounted for in analysis. Statistics, tables, figures and descriptions will focus on relevant gender differences that came up in the course of the project. Findings from this analysis will be included in the final publication. As qualitative data are being collected, the specific impact of the intervention on both male and female participants can be explored in depth. Interviews will allow participants to discuss aspects that are of importance to their own lives, which may or may not be specific to gender. Recruitment will not be gender-balanced, as this is not reflective of the population demographic. Groups (control/intervention) will be balanced, and a representative population will be recruited, if possible.

#### Recruitment

Occupational therapists will be recruited through professional body email (e.g. the Association of Occupational Therapists of Ireland) and through notification on the MS Ireland website, health professionals’ email list and the bi-annual *MS Ireland* research e-zine. The use of snowball sampling will follow, in which occupational therapists may inform other potentially interested occupational therapists of the trial. People with MS will be recruited through trial advertisement in relevant newsletters (e.g. the monthly *MS Ireland* newsletter), on websites offering information and services to people living with MS (e.g. MS Ireland), via recruited occupational therapists as well as through posters and information leaflets posted in relevant clinics (e.g. neurology, general practitioner, primary care clinics, physiotherapy) around the Republic of Ireland. Advertisements will also be posted in any relevant publications, radio, discussion boards and fora, as well as through social media. All individuals interested in participating will self-select through contacting the researchers by phone or email. Informed consent will be obtained and eligibility assessed prior to participation.

#### Randomisation

People with MS will be assigned to occupational therapists, based on geographic location, and the occupational therapists will be randomly assigned to one of two study arms (i.e. COB-MS delivery or wait-list control), using 1:1 allocation, via randomised block permutation. Notably, cluster randomisation was the most appropriate randomisation strategy for this trial, given the potential for contamination in an individually randomised trial. A web-based clinical trial randomisation service will be used (Sealed Envelope). An independent, unblinded researcher will conduct the randomisation, having been provided information regarding sequence and type of randomisation to be used (as described above) by the statistician (who is not involved in participant allocation). Participants will be informed of their allocation by an unblinded research assistant, through post and phone call. Participants’ details will be passed to their allocated occupational therapists to initiate contact and the intervention.

#### Inclusion and exclusion criteria

Occupational therapists will be eligible to participate if they meet the following inclusion criteria for the study: (1) currently working in Ireland as an occupational therapist; (2) registered with Health and Social Care Professionals Council, Ireland; (3) have experience working with people with MS; and (4) can commit to the requirements of the study. Though there are no exclusion criteria for occupational therapists (apart from not meeting the inclusion criteria), if more occupational therapists express an interest in participating than are required, selection criteria will be applied to prioritise occupational therapists based on: (1) geographic proximity to participants with MS; (2) experience of conducting group-based interventions; and (3) experience working with those with cognitive difficulties.

People with MS will be eligible to participate if they meet the following inclusion criteria for the study:

1. Aged 18 years of age or older
2. Fluent in written and spoken English
3. Have a diagnosis of multiple sclerosis (consistent with the McDonald Criteria for the Diagnosis of Multiple Sclerosis [29])
4. Cognitive difficulties, as shown by a score of > 22 on the Multiple Sclerosis Neuropsychological Screening Questionnaire (MSNQ) [30]
5. Are clinically stable (i.e. not having an active relapse)
6. Can provide informed consent
7. No neurologic history other than MS, including evidence of current dementia
8. No history of major depressive disorder, schizophrenia or bipolar disorder I or II
9. No history of diagnosed substance use or dependence disorder
10. Not currently undergoing any other form of cognitive rehabilitation
11. Living in the community.

The exclusion criteria for people with MS are: (1) cognitive impairment that would affect reliable participation or capacity to give informed consent; (2) being incarcerated or institutionalised; and (3) significant neurological condition or organic brain damage (unrelated to MS).

### Measures

#### Primary outcome

*Goal attainment scaling* [31] allows participants to set meaningful goals relating to daily life which can be measured in a systematic way. This technique has shown excellent inter-rater reliability (*r* = 0.95) and construct validity (*r* = 0.92) in cognitive rehabilitation [32]. Goal attainment scaling shows reliability, validity and sensitivity when utilised within a population of adults and older people [33]. The primary endpoint will be at the 12 weeks follow-up.

#### Secondary outcomes

Various tests and measures are used to evaluate the secondary outcomes. They are listed as follows:

- *Symbol Digit Modalities Test (SDMT)* [34]. Within the *Minimal Assessment of Cognitive Function in Multiple Sclerosis (MACFIMS)* [35], the SDMT appears to be the strongest predictor of future cognitive decline, thus supporting its sensitivity and construct validity [36]. Similarly, a meta-analysis [37] found the SDMT to have the strongest correlation (*r* = 0.71) with several assessments of processing speed. Research has shown excellent test-retest reliability (*r* = 0.97) at 2-week [38] and 4-week [39] retest intervals for individuals with MS.
- *California Verbal Learning Test II (CVLT-II)* [40]. The CVLT-II is part of the MACFIMS [35], an established battery of assessments on the fundamental domains of cognition to be assessed in MS. The inter-rater reliability of the CVLT-II ranges from 0.80 to 0.96 [40]. Correlation coefficients for the CVLT-II and CVLT in recall and learning variables range from 0.72 to 0.80, supporting its construct validity [40].
- *Trail Making Test (TMT)* [41]. The TMT has excellent construct validity, with correlation coefficients ranging from 0.73 to 0.90 with other measures such as the Wechsler Adult Intelligence Scale – III [42, 43]. Inter-rater reliability for the TMT is also high, *r* = 0.96 [44].
- *Brief Visuospatial Memory Test-Revised (BVMT-R)* [45]. Similar to the CVLT-II, the BVMT-R is part of the MACFIMS. The inter-rater reliability of the BVMT-R ranges from 0.96 to 0.97, and test-retest reliability coefficients range from 0.60 to 0.84 [45].
- *Everyday Memory Questionnaire-Revised (EMQ-R)* [46]. Research by the authors revealed a Cronbach’s alpha of 0.91 for the EMQ, showing good internal reliability. Strong discriminatory properties were also evident between the neurological and control groups. The researchers then examined the correlations between the EMQ and the EMQ-R, and found that both measures were highly correlated (MS *r* = 0.98; stroke *r* = 0.94; healthy control *r* = 0.97), supporting the EMQ-R as a reliable and valid measure for use in a population of MS.
- *Everyday Memory Questionnaire Revised-Relative* [46]. Participants will be given the option of having a family member complete the EMQ-R to provide a proxy rating of function and potential change. This was added as an optional measure based on public and patient consultation.
- *Generalised Self-Efficacy Scale (GSES)* [47]. The GSES aims to predict participants’ ability to cope with daily difficulties and adaptation to any stressful life events [48]. Cronbach’s alphas ranged from .76 to .90 across 23 countries, with the majority in the high .80s.
- *Modified Fatigue Impact Scale* [49]. This scale is a self-report measure of the impact of fatigue on the participant’s life. It examines physical, cognitive and psychosocial impacts of fatigue. It has a Cronbach’s alpha of .81 and discriminates between fatigue seen in MS and that seen in other conditions.
- *Multiple Sclerosis Quality of Life-54* [50]. This instrument measures health-related quality of life via 54 generic MS-specific items. It has population-based normative data available and both good internal validity and test-retest reliability.
- *General Health Questionnaire-12 (GHQ-12)* [51]. The GHQ-12 is a well-validated and standardised 12-item measure of general levels of distress, which can utilise a 4-point Likert score, with higher scores on the scale indicating higher distress. The GHQ is among the most treatment-responsive measures of psychological distress in MS [52, 53].

#### Screening

The Multiple Sclerosis Neuropsychological Screening Questionnaire [30] is a self-report measure, consisting of 15 items that assess cognitive functioning during daily activities in people with MS, regarding various domains, including attention, processing speed and memory. The MSNQ will be administered as a screen for eligibility. The inclusion criterion for cognitive difficulties will be determined in part by a score of > 22 on the MSNQ.

#### Demographic and clinical information

Participants will be asked to provide details regarding their age, gender, nationality, residence, education and health, as well as occupational and marital status. These details will be used for the purposes of correlational analysis and interpretation of the clinical data. Furthermore, programme attendance (i.e. 0–8) and home-based review activity completion (i.e. 0–7) will be assessed to examine programme adherence and engagement. Moreover, acceptability of measures will be rated by participants, and rates of recruitment, consent and retention will also be analysed and evaluated.

#### Programme and occupational therapist rating

Participants will be asked to rate the acceptability of each COB-MS session, as part of their home-based review activities, on a weekly basis. At the end of the COB-MS programme, participants will also be asked to rate their occupational therapists, via a 6-point Likert scale, on six areas relevant to the delivery of the intervention, as a means of controlling for occupational therapist performance. These results will be anonymous, the data will be pooled and the occupational therapists will be aware of this in advance.

### Interventions

#### COB-MS (experimental) condition

COB-MS is a personalised, patient-centred, occupational therapy-based cognitive rehabilitation intervention aimed at improving daily life functioning for people with MS who are experiencing cognitive difficulties. The programme consists of eight sessions (two individual and six group-based), over a 9-week period. COB-MS focuses on goal setting and attainment through managing the demands of employment and daily life via education, remediation and adaptation, using compensatory strategies and routines that can be integrated into daily contexts, while recognising the impact of emotion, motivation and other non-cognitive functions. Session-by-session content details are outlined in Table 1.

**Table 1:** Overview of COB-MS [22]

Session, week and format	Summary of COB-MS content
Session 1, week 1, individual	***Focus on You*** • Initial meeting with the occupational therapist with briefing on what will be involved in the COB-MS • Goal-setting with the person with MS on occupations that they wish to target
Session 2, week 2, group	***You and Your Cognition*** • Session will deal with education on the brain and the different areas of cognition • Discussion on how MS can impact cognition and commonly affected areas: memory, information processing, attention, problem-solving and new learning • Some discussion on the impact of cognitive difficulties on day-to-day occupations
Session 3, week 3, group	***You, the Centrepiece*** • How the cognitive difficulties affect you • What changes can be made by you as a person • What can we learn that can help? • Examples include internal strategies, challenging negative thinking, getting organised, using a mental blackboard, improving sleep, managing stress and mood
Session 4, week 4, group	***You, the Person*** • What changes can be made by you as a person • What can we learn that can help? • Further exploration of strategies
Session 5, week 5, group	***Your Environment*** • How does the environment impact cognition? • What can we change that might help? • Examples include external memory strategies, using bullet points, managing distraction, seeking help, managing and prioritising your workload/household duties, exercise, impact of other factors on cognition
Session 6, week 6, group	***Focus on Doing*** • How are our occupations and daily life affected? • What we can do to help: integrate what has been covered to date and strategies that might be helpful • Give clear examples of how to adapt or remediate occupations
Session 7, week 7, group	***Seeking New Challenges*** • Seeking new challenges • Setting goals for yourself • Keeping motivated, maintaining progress and adapting • Group conclusion and debrief
Session 8, week 9, individual	***Testing the Application*** • Review goals and strategies used • Set new goals if appropriate, plan for future • Signpost to groups and services • Debrief and summary

COB-MS will primarily be conducted in a community setting (e.g. occupational therapist’s practice or local community centre), though two individual sessions will be conducted in the participants’ own homes (or a convenient location). In total, the COB-MS group will receive approximately 2 h of one-on-one occupational therapy as well as approximately 6 h of group-based occupational therapy focused on cognitive rehabilitation. Each group session consists of a theory/background discussion regarding different aspects of cognition (≈ 10 min), related strategies (≈ 15 min), application to participants’ own life and goals (≈ 20 min) as well as opportunities to practice such evidence-based strategies (≈ 15 min). Session 1 involves an initial visit with an occupational therapist who introduces the programme and helps the participant set personal goals.

Sessions 2–7 are once-weekly group sessions with a small group (i.e. seven to nine participants with MS). Session 8 is a final, individual session that takes place 2 weeks after the last group session. Acceptability interviews (not part of the treatment) will take place within 2 weeks of intervention completion in order to control for cognitive difficulties affecting recall.

People with MS will be provided with a participant handbook (125 pages in length) that includes programme content with each session summarised, as well as space for in-group and home-based review activities. At the end of the intervention, participants can keep the handbooks and are encouraged to review them regularly. Qualified occupational therapists will receive COB-MS training and will be provided with a facilitator handbook, as well as access to an interactive, resource-infused companion website and ongoing supervision by phone, email or in person at their workplace, if requested (e.g. session-specific supervision). A sample of two COB-MS sessions, per occupational therapist, will be audio-recorded and compared for intervention fidelity, with permission from the occupational therapists. In order to reduce the chance of contamination, occupational therapists will be asked to declare that they will not share their knowledge or the manual/resources with non-trained occupational therapists and will not use COB-MS methods outside of the trial.

#### Control condition

Participants randomised to the treatment as usual, wait-list control arm of the study will not receive the COB-MS during the trial, but will be provided the programme at the end of the data collection period. They will receive standard clinical care. They will be contacted by an unblinded research assistant to explain that they have been allocated to the wait-list control group. Controls will be assessed at the same time points as the intervention arm.

**Fig. 2:**
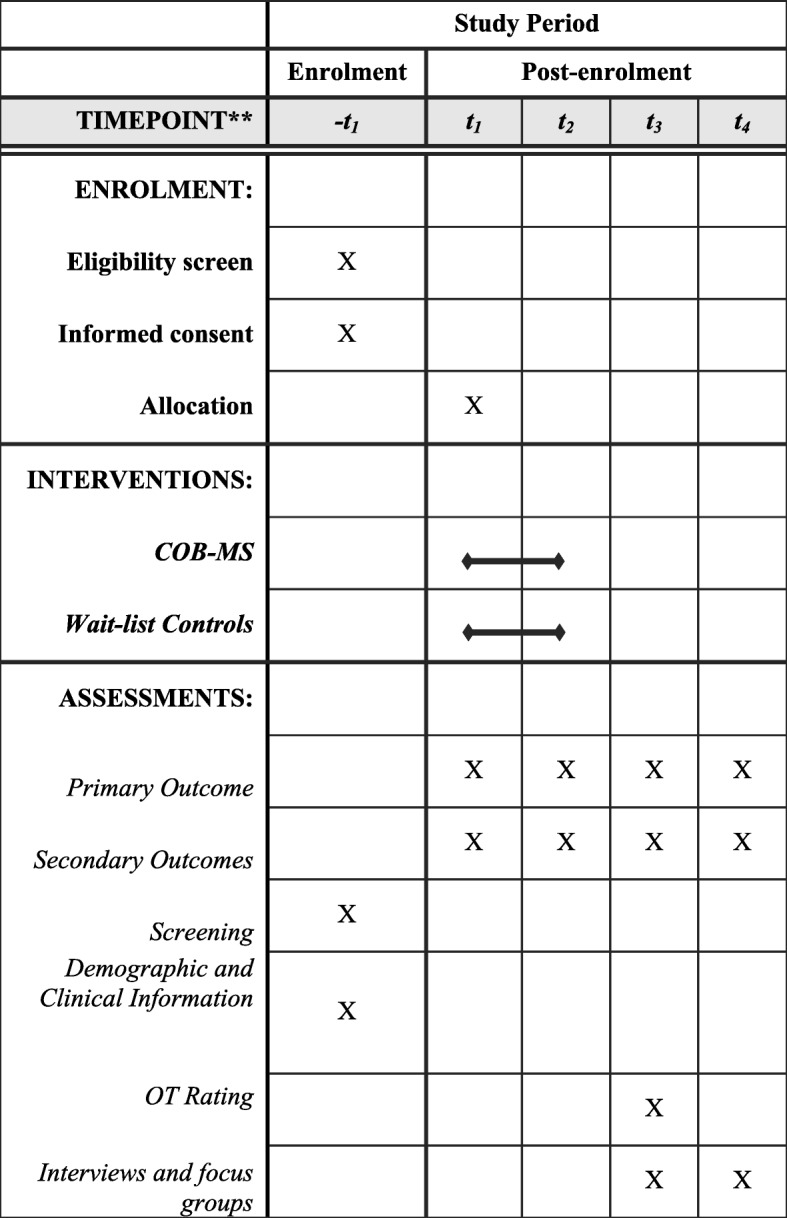
Standard Protocol Items: Recommendations for Interventional Trials (SPIRIT) figure for the schedule of enrolment, interventions and assessment

Because services available to people with MS vary, we will be recording occupational therapy and cognitive interventions received by participants during the study period. We will also gather data on the national picture for cognitive interventions in MS. See Fig. 2 for the schedule of enrolment, interventions and assessment.

#### Fidelity assessment

Fidelity will be assessed to ensure adherence to the COB-MS intervention delivery. Recommendations from the National Institute of Health Behaviour Change Consortium [54] will be used as a framework to increase fidelity in five domains: Study Design, Training, Delivery, Receipt, Enactment. Strategies that will be used include training occupational therapists in a standardised way as well as supporting them throughout the intervention delivery, providing occupational therapists and participants with MS with a handbook that details the content of the intervention. A record of the sessions will be kept by occupational therapists, and participants with MS will record their experiences of each session. Occupational therapists will keep a record of the intervention session content, length and other important information after each session, including attendance and home activity adherence. Two sessions per occupational therapist will be recorded to ensure adherence to the COB-MS programme. Feedback will also be collected through interviews and focus groups with the occupational therapists and participants with MS. Maintaining high levels of treatment fidelity will be a key focus in this feasibility trial [55], so we can be confident about the reliability of the results that come from the trial [56].

### Statistical analysis

Data analysis and reporting will follow 2010 CONSORT guidelines. The feasibility outcomes, recruitment rate, acceptability of COB-MS, rate of unblinding, retention rate and randomisation methods will be reported descriptively and narratively. Analysis will take place once all data have been collected. Means and standard deviations (or medians and interquartile range [IQR] as appropriate) will be used for continuous variables, and counts and percentages for categorical outcomes. The retention rate will be estimated using a 95% confidence interval (CI). Estimates (mean and standard deviations) of the primary outcome variable (i.e. goal attainment scaling), at week 12, will inform sample size calculations of a future definitive trial. Data resulting from primary and secondary outcome measures will be evaluated, per protocol, in terms of preliminary efficacy through a series of 2 (condition) × 4 (time) mixed (multivariate) analyses of variance as well as through correlational analysis. No analysis of health economics or cost-effectiveness will take place in this feasibility study.

### Embedded qualitative component

A descriptive qualitative approach based on the work of Sandelowski [57] will be used. Semi-structured face-to-face interviews will be conducted with participants with MS regarding their experiences and perceptions of taking part in the COB-MS. These data will be collected from 25% (*n* = 15) of those participating in the intervention arm. Purposeful sampling using maximum variation will be used to include, for example, location, age, gender and MS type. Online focus group interviews will be conducted with all occupational therapy participants (*n* = 8) following completion of COB-MS to capture their experiences and perception of delivering the programmes as well as the feasibility for occupational therapists to potentially incorporate COB-MS into their future practice. Participants who decline to participate will be invited to partake in a one-on-one telephone interview to explore reasons for declining and to inform the recruitment processes for any future definitive trial. Data will be analysed using thematic analysis as outlined by Braun and Clarke [58]. The analysis will be an iterative and recursive process, characterised by continual reading and re-reading of the data, data coding and thematic identification. In addition, the feasibility of including a member of the Patient and Public Involvement (PPI) team (see below) to participate in the data analysis process and the potential benefits of the same on study outcomes will be explored. Rigour will be protected by employing the criteria described by Lincoln and Guba [59], and the qualitative data analysis software NVivo will be used to support the analysis.

### Blinding

The study will be single-blinded, as participants will know to what arm of the study they have been allocated, given the wait-list nature of the treatment-as-usual control condition. The research assistant collecting the data will be blind to participant allocation. Structures will be in place that will increase the likelihood of blinding [60], such as concealment of group identity to the research staff administering assessment; staff recording of to which group they thought the participant had been allocated (and their degree of certainty); and reporting of unblinding rates. Unblinding will only be permissible in exceptional circumstances when knowledge of allocation is absolutely essential. In these circumstances blinding will still be maintained as far as possible—the actual allocation will not be disclosed to research staff not involved in the unblinding or to others who had previously been blinded to allocation. All code breaks will be reported on the case report form, and the reasons why will be provided.

### Patient and participant involvement

Patient and Public Involvement (PPI) is an effective means of enhancing the likelihood of a successful trial by involving people with lived experience of a particular condition (i.e. MS) as partners throughout the research process [61, 62]. Consistent with PPI practice, this trial has a PPI member as a member of the research team for the entire duration of the trial. There are two PPI members on the Trial Steering Committee, and an external PPI consultation group has been convened to discuss issues, outcome measures and recruitment material. We will disseminate findings and experiences through lay media (e.g. social media, newsletters and other public fora) and knowledge translation events in the community. Given the current underdevelopment of the PPI evidence base, appropriate, relevant processes will be developed to evaluate and report the impact of PPI activities [63], consistent with established protocols (e.g. Guidance for Reporting Involvement of Patients and the Public – 2, Long-form) [64].

### Data management and monitoring

A FAIR data management plan [65] will be used for this COB-MS project, which ensures that all data are *findable*, *accessible*, *interoperable* and *reusable*. For the detailed data management plan (DMP) the DMP Online Digital Curation Centre checklist will be used to guide the creation of the full DMP. This DMP will outline how data outputs from the COB-MS project will be managed and shared under the following headings:

1. Data outputs from the research: what will be collected and processed
2. Handling of research data during and at the end of the project
3. When data will be shared and what data types will be shared
4. Where data will be made available
5. How data will be accessed
6. Safeguarding research participants
7. Intellectual property
8. Resources.

Data will be entered onto an electronic database provided by the Health Research Board (HRB) Clinical Research Facility. Research staff entering data will have an individual log-in that will allow access to blinded or unblinded sections of the database, as appropriate. Accuracy of data entry will be audited as detailed in the data management plan.

Data will be shared at the following locations:

1. Irish Social Science Data Archive
2. Irish Qualitative Data Archive.

Confidentiality of all data and individual results will be protected at all times, and anonymisation will be used throughout the study. Names or other personal identifiers will be securely stored separately from other study data/records identified by code/pseudonym. The statistician will analyse cleaned, depersonalised data. Blinded researchers, including the statistician, will only have access to cleaned, depersonalised data sets. With respect to qualitative data, audio files will be destroyed upon transcription (i.e. yielding anonymous data). Participants will be aware of and have consented to these processes in advance of participation.

### Trial oversight

A Trial Management Group has been established and is responsible for ensuring timely delivery of trial activities. The responsibilities of this group will be in the overseeing of the management and running of the COB-MS trial.

The trial will be monitored by an independent Trial Steering Committee (TSC) with an independent chair. The role of the TSC is to provide oversight of the trial and ensure that the trial is conducted in accordance with the principles of good clinical practice and relevant regulations. This committee will meet three times a year, and its members are independent of the trial. The principal investigator (PI) will be on the TSC as a non-independent member and will highlight any particular issues for discussion and requests for guidance at meetings. Any issues that might impact the trial will be discussed by the committee. The TSC will focus on the progress of the trial, adherence to the protocol and participant safety.

For this trial, given that the risk is considered to be low /relatively small, the TSC will assume the role of the Data Monitoring Committee (DMC). The DMC terms of reference have been drawn up. This document outlines stopping rules and any planned data analyses. All members of the DMC will be independent of the trial. They will be made up of two to four members of the TSC and will include a statistician, clinician and independent chair.

### Progression criteria

A traffic light system—green (go), amber (amend) and red (stop)—which allows for modification will be used [66], in consultation with the TSC. The key areas of risk are included in the criteria: trial recruitment, protocol adherence and outcomes. Criteria in the Acceptance Checklist for Clinical Effectiveness Pilot Trials (ACCEPT) [67] will also be used to evaluate progression. They will examine (1) feasibility and appropriateness of the trial design; (2) feasibility and appropriateness of the mechanics, management and safety of interventions; (3) acceptability and efficiency of implementing the research procedures. Trial components, exemplar monitoring methods and exemplar outcomes are included in the checklist. Transparent reporting will be made around the decision-making process for stopping, amending or proceeding to a main trial, as recommended by Avery et al. [66].

### Dissemination

Findings regarding feasibility and efficacy—regardless of direction, significance or magnitude of effect(s)—will be submitted for publication in peer-reviewed journals and presentation at both national (Ireland) and international conferences. This study’s knowledge exchange plan also includes accessible outlets of dissemination for lay audiences, such as through PPI-oriented national meetings and other local level presentations and fora, social media and also NUI Galway and MS Ireland communications, with an aim of targeting people with MS, the research community, funding bodies, healthcare providers and healthcare policy. Study results will be submitted for appropriate dissemination within 6 months of final data collection.

## Discussion

There is currently no standardised approach for the rehabilitation of cognition in MS. Were this program successful, it would have a significant clinical impact on the way in which healthcare professionals work with people with MS who report cognitive difficulties. This research is especially timely given the clear need by patients for self-management tools for symptoms in MS, as intervention for cognition is a priority for Irish people with MS [21, 22]. The impact of this study lies in the essential role that it plays in establishing the feasibility of this cognitive intervention for people with MS. This trial is an essential step in ensuring that the most robust and well-designed methodologies are used to assess the COB-MS effectiveness, as recommended by previous research and reviews in the area. If effective, the COB-MS has strong potential for immediate use in clinical practice, as it requires few resources other than group facilitator training. It intervenes in a group setting, allowing for resources and practitioner time to be used efficiently. It is likely to directly contribute to improved patient care, as healthcare providers will have an evidence-based programme that is specific to the difficulties seen in MS.

### Trial status

The protocol is version 1.0 (4 December 2019). Recruitment start was in November 2019; completion of recruitment should be in June 2020.

## Data Availability

Data sharing is not applicable to this article, as no data sets were generated or analysed for this protocol. Data and materials will be made available from the trial once conducted.

## Abbreviations

BVMT-R: Brief Visuospatial Memory Test-Revised
COB-MS: Cognitive Occupation-Based programme for people with Multiple Sclerosis
CVLT-II: California Verbal Learning Test II
EMQ: Everyday Memory Questionnaire
MACFIMS: Minimal Assessment of Cognitive Function in Multiple Sclerosis
MSNQ: Multiple Sclerosis Neuropsychological Screening Questionnaire
PPI: Patient and Public Involvement
RCT: Randomised controlled trial
SDMT: Symbol Digit Modalities Test
TMT: Trail Making Test
TSC: Trial Steering Committee
